# Enhancing effect of *Tiliacora triandra* leaves extract on spatial learning, memory and learning flexibility as well as hippocampal choline acetyltransferase activity in mice

**Published:** 2018

**Authors:** Thong-asa Wachiryah, Laisangunngam Hathaipat

**Affiliations:** 1 *Animal Toxicology and Physiology Specialty Research Unit (ATPSRU), Physiology Division, Department of Zoology, Faculty of Science, Kasetsart University, Bangkok, Thailand*; 2 *Department of Zoology, Faculty of Science, Kasetsart University, Bangkok, Thailand*

**Keywords:** Choline acetyltransferase, Spatial learning, Learning flexibility, Hippocampus, Tiliacora triandra, Morris water maze

## Abstract

**Objective:**

The present study investigates the effect of *Tiliacora triandra* leaf extract on spatial learning, memory, and learning flexibility as well as hippocampal choline acetyltransferase (ChAT) activity in mice.

**Materials and Methods:**

Thirty male ICR mice were randomly divided into three groups including 10% Tween 80, *T. triandra* 300 mg/kg and *T. triandra* 600 mg/kg. All administrations were done orally for 18 consecutive days. Spatial learning, memory and learning flexibility were assessed using the Morris water maze. ChAT activity and hippocampal neuronal cell number were assessed by immunohistochemistry and histological methods, respectively.

**Results:**

The results demonstrated that *T. triandra* leaf extract (300 and 600 mg/kg) significantly enhances spatial learning and learning flexibility. Only 300 mg/kg of* T. triandra* significantly improved the spatial memory. The hippocampal ChAT activity and total hippocampal cell number were significantly increased in *T. triandra*-treated groups.

**Conclusion:**

The present study indicated that *T. triandra* leaf extract improves the spatial learning, memory and learning flexibility, exerts neuroprotective effects on hippocampal neurons and maintains ChAT activity in this brain area.

## Introduction

*Tiliacora triandra* or Yanang is a plant commonly used in northeast of Thailand. It belongs to the family of Menispermaceae, possesses green oval leaves and yellowish flowers and is commonly found in the evergreen forests. Thai traditional medicine uses this plant against fever and alcohol intoxication. *T. triandra* has anti-bacterial, anti-malarial and anti-inflammatory properties (Saiin and Markmee, 2003 ▶; Sureram et al., 2012 ▶). Leaves of *T. triandra* serve as a natural anti-oxidant source because it has high levels of beta-carotene, condense tannin, triterpene, flavonoids and saponins (Boonsong et al., 2009 ▶). Recently, it was shown that *T. triandra* reduces the risk of cancer (Kaewpiboon et al., 2014 ▶). *T. triandra* also has an inhibitory effect on acetylcholine esterase (AChE), the key enzyme of breaking down acetylcholine (ACh) into choline and acetic acid. Considering the anti-oxidant and AChE inhibitory properties of this plant, it might be beneficial to cognitive abilities (Ingkaninan et al., 2003 ▶). 

Acetylcholine is an important neuromodulator involved in a wide variety of brain functions such as cognition, attention, consciousness and motor functions. Learning and memory are important cognitive functions of the brain that are related to the modulatory role of septohippocampal ACh (Ch1 and Ch2) pathway which is the connection of medial septum (MS) cholinergic neurons and hippocampus via the fimbria-fornix (Giovannini et al., 1997 ▶; Oda, 1999 ▶; Pepeu et al., 1990 ▶; Pinto et al., 2011 ▶). The hippocampus and its trisynaptic circuit plays a critical role in learning and memory in both acquisition and retrieval processes (Andersen et al., 1971 ▶) and involve the cholinergic system (Deiana et al., 2011 ▶). Spatial memory is one of essential forms of higher cognitive processing in mammals in which the hippocampal function is of crucial importance. Spatial memory is the capacity to record information about one’s surrounding environment and the spatial orientation. This type of memory can be tested in rodents using the Morris water maze (MWM) which was developed by Richard G. Morris since 1981 (Morris, 1984 ▶). Acquisition of spatial tasks correlates with the hippocampal ACh release (McIntyre et al., 2003 ▶) and particularly the place cell firing during and after learning (Goonawardena et al., 2011 ▶). Moreover, impairment of hippocampal ACh release was found in parallel with spatial memory deficit in mice with basal forebrain cholinergic degeneration (Laursen et al., 2014 ▶). Labelling of choline acetyltransferase (ChAT), the most specific indicator of the functional state of cholinergic neurons, revealed the correlation of ChAT changes and spatial learning impairment (Gallagher et al., 1990 ▶). Therefore, the aim of the present study was to investigate the neurotonic effect of *T. triandra* leaves extract on spatial learning, memory, and learning flexibility as well as dorsal hippocampal ChAT activity in mice. 

## Materials and Methods

**Plant collection and extraction**

*T. triandra *was obtained from Ladyao, Jatujak (District), Bangkok, Thailand. The classification of plants was confirmed by a plant taxonomist of ASESRU, Faculty of Science, Kasetsart University. Air-dried *T. triandra* leaves were powdered and extracted using 95% ethanol (EtOH) in Soxhlet extractor for 20 hr. Leaves crude extract was filtered and concentrated using a rotary vacuum. This process was repeated three times to obtain a dark-green crude extract which was stored in an air-tight bottle at 4^o^C until used.

**Animals**

Thirty male ICR mice (*Mus musculus*), weighting between 40–50 g, were used. They were obtained from the National Laboratory Animal Centre, Mahidol University, Salaya, Nakornprathom province. Mice were housed under 12hr/12hr light-dark cycles with well-controlled temperature (23±2^o^C) and humidity (55±5%) and had free access to standard food pellets and reverse osmosis (RO) water. This research was conducted in accordance with internationally accepted principles for laboratory animal use and care and the experimental protocol was approved by Animal Ethics Committee, Kasetsart University Research and Development Institute (KURDI), Kasetsart University, Bangkok, Thailand (ID#OACKU 00158).

**Experimental protocol**

In brief, mice were randomly divided into three groups including mice that received 10% Tween 80, mice that were treated with *T. triandra* 300 mg/kg and mice that were treated with *T. triandra* 600 mg/kg (n=10 in each group). The treatments were orally administered via gavage and continued for 18 days. The vehicle was 10% Tween 80. *T. triandra* leaves extract 300 and 600 mg/kg were prepared from a stock concentration of 300 mg/ml of *T. triandra* leaves extract prepared in 10% Tween 80. 

**Cognitive tests using the Morris water maze**

The Morris water maze is a plastic pool (150 cm in diameter and 50 cm tall), filled with tap water (25^o^C) with 40 cm depth. Cognitive tests were started on day 7 after oral administration. Prior to spatial learning tests, sensorimotor evaluation was done in order to assess visual and motor abilities by the visible platform paradigm. Briefly, visible platform was placed above the water surface to be easily seen by the animals. All mice were given four trials to swim, search, and sit on the visible platform. The maximum time for each trail was 120 sec. The swimming speed of mice in each group was compared for sensorimotor evaluation. On the following day, spatial learning was tested on five days and defined as the acquisition trial. Briefly, the pool was divided into four quadrants: northeast (NE), northwest (NW), southeast (SE), and southwest (SW). The hidden platform was placed under the water surface in the center of the NE quadrant (the target quadrant of acquisition trial). A variety of visual cues were placed around the pool. Mice were continuously given four trials a day with 120 sec per each trial. When the acquisition trial was completed, the probe trial was delivered in order to determine spatial memory by hidden platform was removed from the target quadrant, and mice were allowed to swim for 60 sec and the time spent in each quadrant was recorded and further converted to percentage of time spent in each quadrant to evaluate the memory capacity. 

On the following day, learning flexibility was assessed in the reversal trial. The only difference from the acquisition trial was the moving of hidden platform to the opposite quadrant (SW). In order to assess learning flexibility that how rapidly the mice switch their search strategies to the new goal position (Thong-asa et al., 2017 ▶; Vorhees and Williams, 2006 ▶; Weitzner et al., 2015 ▶). 

**Histological analysis**

After finishing all cognitive tests, all mice were scarified by an intraperitoneal overdose of sodium pentobarbital (>60 mg/kg). They received intracardiac perfusion using 0.9% normal saline solution (NSS), followed by 4% paraformaldehyde (PFA) in 0.1 M phosphate buffer saline (PBS) (pH 7.4). The brains were removed and stored in 4% PFA in 0.1 M PBS (pH 7.4) for 24 hr before being processed and embedded in paraffin block. 

**Cresyl violet staining**

Brain sections (5-µm thickness) of the dorsal hippocampus at bregma–1.98 were collected (Paxinos and Franklin, 2008 ▶). These brain sections were stained with 0.1% cresyl violet for histological analysis. In brief, after deparaffinization and rehydration, brain sections were soaked in 70% EtOH, distilled water and stained with 0.1% cresyl violet for 30 sec. Brain sections were dehydrated with 95% EtOH, xylene followed by mounting with cover glass. 

**ChAT activity assessment using immunohistochemistry**

Brain sections were kept in hot-air oven at 60^o^C, overnight, deparaffinized and rehydrated in three changes of xylene followed by treatment with 100% and 95% EtOH and running tap water for 5 min. Antigen epitope was retrieved in citrate buffer pH 6.0 (S1699, Agilent, USA) for 3 min of high power and 10 min of 30% power in microwave. After a cool-down period of 20 min, brain sections were washed with running tap water for 5 min then blocked with peroxidase blocking (S2023, Agilent, USA) for 30 min followed by another wash with running tap water for 5 min. Brain sections were washed in washing buffer (S3006, Agilent, USA) before incubated with rabbit anti-ChAT polyclonal antibody (1:500) at 4^o^C for 48 hr. After washing with washing buffer, brain sections were incubated with EnVision FLEX/HRP visualization reagent SM802 for 30 min, washing and developing color in SM803 and DM827. After 5 min washing with running tap water, brain sections were dipped in 95% and 100% EtOH, xylene and sealed with cover glass. 

**Image analysis**

Five brain sections were collected from each mouse (the space interval was 125 µm). Images were captured by Olympus Tg300 microscopy. The areas of interest were the cornus ammonis (CA) 1, 3, and dentate gyrus (DG) of the dorsal hippocampus. Viable cells were counted in all captured images at 400X magnification. Viable cells were characterized by a visible nucleus and nucleolus with light purple color of cytoplasm. The diameters of cells ranged between 15 and 35 µm in the CA1 and CA3 regions and from 9 to 25 µm in the DG region. Viable cells were counted in a blind fashion by two investigators using the UTHSCSA Image Tool 3.0. 

The ChAT labeling density was analyzed by NIH Image J. In brief, all captured images of CA1, CA3 and DG at 400X magnification, were converted to a binary image (black & white color) and threshold adjustment was manually done before analysis. ChAT labelling density was represented as the percentage (%) of area (Farkas et al., 2004 ▶; Wakita et al., 2002 ▶).

**Statistical analysis**

The data was expressed as mean ± standard error of mean (SEM). Escape latencies, determination of spatial learning and learning flexibility were analyzed by repeated-measures ANOVA followed by Fisher’s *post-hoc* test. Percentage of time spent in the target quadrant, determination of spatial memory capacity, enumeration of viable cells and evaluation of percent area of ChAT labelling were analyzed by one-way ANOVA followed by Fisher’s *post-hoc* test. Statistical significance was accepted when p-value<0.05.

## Results

Animal weights and the organs weights did not vary significantly from week one to week three of the experiment. Sensorimotor evaluation represented by the swim speed (cm/sec) was not different among the groups (Table 1). Administration of *T. triandra* leaves extract and/or 10% Tween 80 had no effect on growth and sensorimotor function.

**Figure 1:**
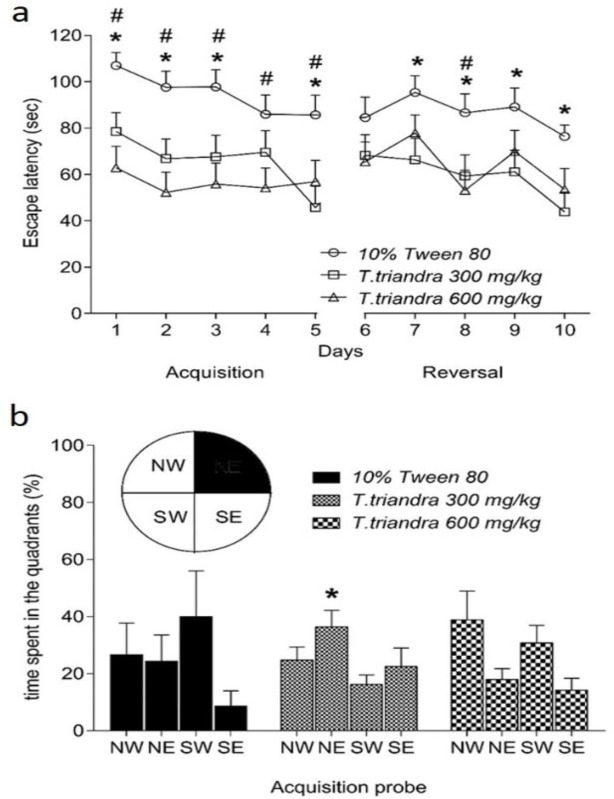
Spatial learning (Day 1-5) and learning flexibility (Day 6-10) represented by the escape latency (a). Spatial memory was tested in the probe trial (b) represented by the percentage of time spent in the target quadrant (northeast; NE). * indicates a significant difference at p<0.05 between *T. triandra* 300 mg/ kg group and 10% Tween 80 group. # indicates a significant difference at p<0.05 between *T.triandra* 600 mg/kg group and 10% Tween 80 group

Spatial learning as assessed by MWM assay, showed that mice treated with *T. triandra* 300 and 600 mg/kg spent less time to find the hidden platform than the control group. Significant differences were found on days 1, 2, 3 and 5 in acquisition phase between the mice treated with *T. triandra *300 mg/kg and those treated with 10% Tween 80 (day 1, p=0.0119; day 2, p=0.0108; day 3, p=0.0135 and day 5, p=0.0010). Also, significant differences were found on days 1–5 in acquisition phase between the mice treated with *T. triandra* 600 mg/kg and those treated with 10% Tween 80 (day 1, p=0.0001; day 2, p=0.0002; day 3, p=0.0006; day 4, p=0.0116 and day 5, p=0.0163) (Figure 1a). Spatial memory capacity that was assessed in probe trial, demonstrated significant enhancement of spatial memory only in mice treated with *T. triandra* 300 mg/kg (p=0.0264) but not in mice treated with *T. triandra* 600 mg/kg as compared to animals treated with 10% Tween 80 (Figure 1b). 

**Table 1:** Body and organ weights (g), sensorimotor evaluation (cm/sec), ChAT labelling density (% area) and viable cells number in the CA1, CA3 and DG of the dorsal hippocampus. The data are presented as mean±SEM

**Parameters **		**Groups**		
		**10% Tween 80**	***T. triandra*** ** 300 mg/kg**	***T. triandra*** ** 600 mg/kg**
**Body weight **(g)		42.857±1.844	42.333±1.453	46.000±4.000
**Brain **(g)		0.629±0.011	0.647±0.030	0.590±0.020
**Liver **(g)		2.303±0.116	2.073±0.217	2.230±0.580
**Kidneys **(g)		0.697±0.044	0.577±0.029	0.640±0.060
**Lungs **(g)		0.266±0.017	0.297±0.062	0.325±0.055
**Heart **(g)		0.269±0.014	0.213±0.023	0.235±0.005
**Testes **(g)		0.339±0.031	0.370±0.015	0.360±0.050
**Swim speed **(cm/sec)		19.854±1.929	23.320±1.972	21.642±1.151
**ChAT density**	**CA1**	11.631±2.102	14.315±1.417	17.671±1.178*
	**CA3**	10.301±2.021	12.385±0.982	12.074±0.959
	**DG**	8.025±1.604	11.866±1.010*	11.566±1.165*
**Viable cells**	**CA1**	548.583±94.197	645.786±43.023	919.846±24.941*
	**CA3**	394.917±68.456	453.143±37.644	450.692±57.304
	**DG**	1419.417±82.716	1629.357±46.567	1453.769±87.798

* p<0.05 shows significant differences as compared to mice treated with 10% Tween 80.

**Figure 2:**
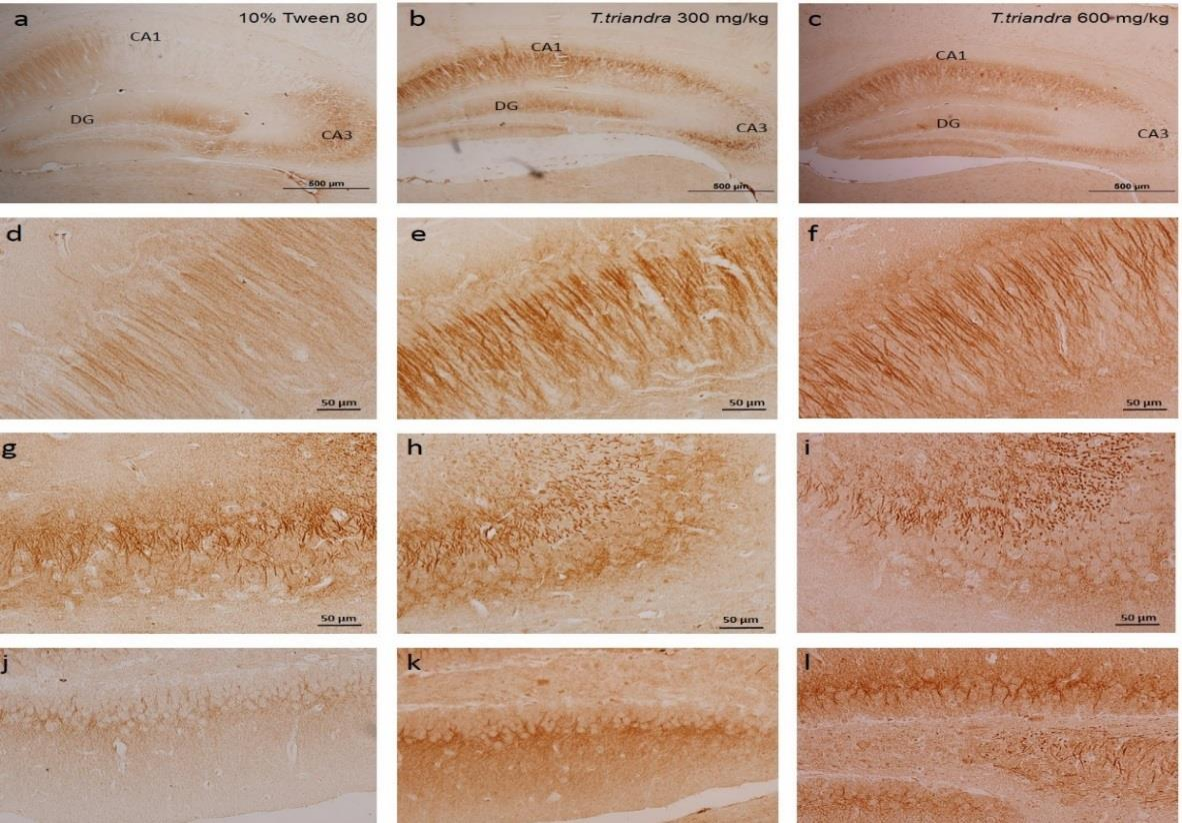
Representative photomicrograph of ChAT immunohistochemistry in the dorsal hippocampus at 40X magnification (a–c) (scale bar: 500 µm). ChAT immunohistochemistry of CA1 (d–f), CA3 (g–i) and DG (j–l) at 400X magnification (scale bar: 50 µm)

Reversal phase demonstrated that mice treated with *T. triandra* 300 mg/kg and mice treated with *T. triandra* 600 mg/kg showed significant enhancements in the learning flexibility. The results of *T. triandra* 300 mg/kg group were significantly different on days 7-10 (day 7, p=0.0135; day 8, p=0.0176; day 9, p=0.0186 and day 10, p=0.0059) from the 10% Tween 80 group while *T. triandra* 600 mg/kg group had significantly different results only on day 8 (p=0.0035) as compared to mice treated with 10% Tween 80. 

**Figure 3:**
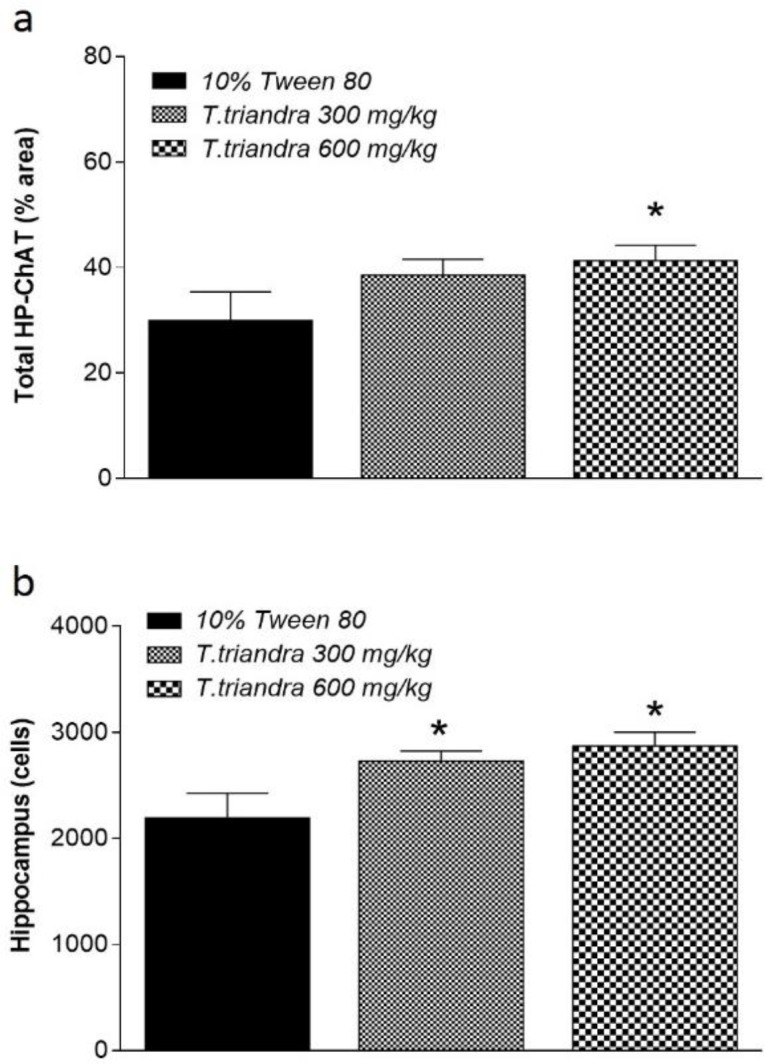
Histogram of total hippocampus (HP) ChAT labeling density (a) and total viable cells number in the dorsal hippocampus (b). * indicates significant difference at p<0.05 as compared to mice treated with 10% Tween 80

ChAT labelling density in the CA1 and DG areas of the dorsal hippocampus was significantly increased in mice treated with *T. triandra* 600 mg/kg (p=0.0110 and 0.0369, respectively) while in mice treated with *T. triandra* 300 mg/kg, it was significantly increased only in DG (p=0.0180) as compared to 10% Tween 80 group (Table 1). Evaluation of total hippocampal ChAT density showed significant increases only in mice treated with *T. triandra* 600 mg/kg (p=0.0348) (Figure 3a). Viable cells number in mice treated with *T. triandra* 300 and 600 mg/kg were higher than that of the vehicle-treated mice in all areas of the dorsal hippocampus. Significant differences were found only in CA1 area in mice treated with *T. triandra* 600 mg/kg (p=0.0001) (Table 1). Enumeration of viable cells in all areas of the dorsal hippocampus demonstrated significant increases in viable cells number in mice treated with *T. triandra* than vehicle-treated mice (p = 0.0039 and p=0.0161 in 300 and 600 mg/kg-treated mice, respectively) (Figure 3b). 

## Discussion

The present study demonstrated that *T. triandra* leaves extract enhances spatial learning, memory and learning flexibility; *T. triandra* leaves extract 300 mg/kg significantly improved both spatial learning and memory but at 600 mg/kg, it did not influence the spatial memory. Dose of *T. triandra* leaves extract impacted difference on spatial cognitions. The present study assessed spatial cognitions in terms of acquiring and retrieving the platform location in the MWM. We found that *T. triandra* 300 mg/kg enhances both acquiring and retrieving spatial cognitions but at 600 mg/kg, it only enhances acquiring process of spatial navigation. The bell-shaped effect on spatial memory of *T. triandra* leaves extract may be related to its polyphenolic content. Polyphenols content of *T. triandra* leaves extract are p-hydroxybenzoic acid, minecoside, flavones glycoside cinnamic acids derivative and monoepoxy-betacarotene (Boonsong et al., 2009 ▶). A report found that p-hydroxybenzoic acid exhibits sedative and hypnotic activities in mice (Holzmann et al., 2014 ▶) and may influence motivation in spatial memory test. Although in the MWM, equal-motivation was present for swim and escape (Vorhees and Williams, 2014 ▶), sedative and hypnotic properties of polyphenolic compounds present in *T. triandra* may reduce these abilities thus resulting in no enhancing effect on spatial memory as observed in the present study. Most polyphenols have bell-shaped dose-response effects (i.e. inducing cellular toxicity at high concentrations but cellular benefits at low concentrations) (Vauzour, 2012 ▶). Considering the effect of *T. triandra* leaves extract on viable cells number in the whole hippocampus, it was found that mice treated with *T. triandra* leaves extract show significantly higher numbers of viable cells compared to mice treated with the vehicle. The difference in cell numbers might involve the promoting of cell survival induced by *T. triandra* leaves extract against stress induction in the hippocampal-dependent task (Xu et al., 2011 ▶). Stress of handling, oral administrations and force swimming in cool water might affect the hippocampal cells. The hippocampus is the target of stress hormones (e.g. glucocorticoid) and it was evidenced that repeated stress caused atrophy of dendrites in CA3 sub-region and both acute and chronic stresses inhibited DG neurogenesis (McEwen, 1999 ▶). The stress-induced neuronal damage may be mediated though sympatho-adreno-medullary system and the hypothalamo-pituitary-adrenal system (de Kloet et al., 2005 ▶). The hippocampus has numerous mineralocorticoid receptors (MR) and glucocorticoid receptors (GR) which lead to its high sensitivity to stressful stimuli, particularly in the CA1 region (Joels et al., 2008 ▶). Activation of these receptors correlated with inducing neuronal damage via several pathways, one of these is oxidative damage (Fontella et al., 2005 ▶). Hence, prevention of the neuronal number reduction in the dorsal hippocampus in the present study may be due to anti-oxidant properties of *T. triandra* leaves extract (Boonsong et al., 2009 ▶; Ingkaninan et al., 2003 ▶; Singthong et al., 2014 ▶). Our previous study showed that *T. triandra* leaves extract can prevent spatial learning, memory and learning flexibility deficit due to DG neuronal death in mice following ischemia/reperfusion (Thong-asa et al., 2017 ▶). In the present study, we found significant increases in ChAT density in this brain area which may be related to enhancing effects of *T. triandra* leaves extract on cognitive functions. Previously, the correlation between ChAT change and spatial learning impairment was reported (Gallagher et al., 1990 ▶); the present study indicated the enhancing effects of *T. triandra* leaves extract on spatial learning, memory and learning flexibility are mediated via its neuroprotective effect on hippocampal neurons and maintaining ChAT activity in this brain area. 
